# Food groups and the likelihood of non-alcoholic fatty liver disease: a systematic review and meta-analysis

**DOI:** 10.1017/S0007114520000914

**Published:** 2020-07-14

**Authors:** Kaiyin He, Yuting Li, Xin Guo, Lu Zhong, Shaohui Tang

**Affiliations:** Department of Gastroenterology, The First Affiliated Hospital, Jinan University, Guangzhou, Guangdong, Peopleʼs Republic of China

**Keywords:** Non-alcoholic fatty liver disease, Liver steatosis, Risk of non-alcoholic fatty liver disease, Diet, Food groups, Meta-analyses, Systematic reviews

## Abstract

Dietary habits have been implicated in the development and severity of non-alcoholic fatty liver disease (NAFLD). Several epidemiological studies attempted to assess the relationship between food groups and the likelihood of NAFLD, but these results were conflicting. The present meta-analysis was conducted to assess the association between food groups and the likelihood of NAFLD. Published literature was retrieved and screened from MEDLINE, Embase and Web of Science. Out of 7892 retrieved articles, twenty-four observational studies (fifteen cross-sectional studies and nine case–control studies) met our eligibility criteria and were finally included in this systematic review and meta-analysis. Consumption of both red meat and soft drinks contributed to a positive association with NAFLD. Inversely, nut consumption was negatively associated with NAFLD. There were no significant influences on the likelihood of NAFLD about consuming whole grains, refined grains, fish, fruits, vegetables, eggs, dairy products and legumes. This meta-analysis suggests that individuals who consumed more red meat and soft drinks may have a significantly increased likelihood of NAFLD, whereas higher nut intake may be negatively associated with NAFLD. Further prospective studies are required to assess the association between food patterns and NAFLD.

## Graphical abstract

**Figure:**
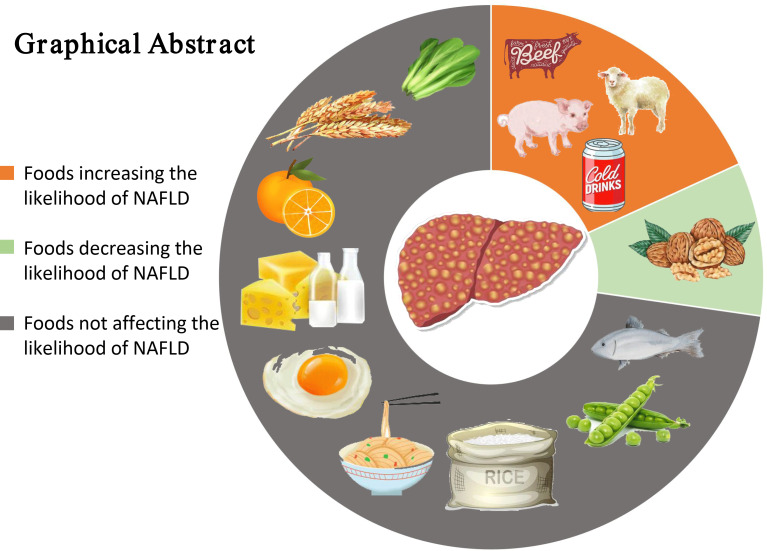

With the rising prevalence of obesity, diabetes mellitus and the metabolic syndrome, non-alcoholic fatty liver disease (NAFLD) has been considered the most common liver disease which affects 20–30 % of the worldwide population^(1)^. NAFLD is characterised by the accumulation of hepatic fat >5 % and not caused by excessive alcohol consumption, use of hepatotoxic medications or other established liver diseases^(2)^. It encompasses a spectrum of liver damage that can progress from simple steatosis to non-alcoholic steatohepatitis, hepatic fibrosis and cirrhosis. Approximately 30 % of patients with simple steatosis progress to non-alcoholic steatohepatitis, which can potentially progress to fibrosis/cirrhosis and eventually lead to hepatocellular carcinoma^(3)^.

Metabolic changes, including insulin resistance and impaired lipid metabolism, have been identified as the molecular pathogenesis of NAFLD^(4)^. NAFLD, which is similar to metabolic diseases such as obesity, inflammation, insulin resistance and type 2 diabetes, is considered to be a liver component of the metabolic syndrome^(5)^. The Western dietary pattern characterised by higher loads of energy content, saturated fat, fructose, sugar-sweetened beverages and refined carbohydrates is associated with weight gain, obesity and more recently with NAFLD^(6)^. Although there is currently no consensus on the pharmacological treatment of NAFLD, the international guidelines recommend that lifestyle modification associated with weight loss should be an integral part of the treatment of NAFLD^(7)^.

Lifestyle modifications include achieving weight loss, increasing physical activity and acquiring a healthy dietary pattern^(8)^. Although weight loss is an important approach for the management of NAFLD, extreme dietary intervention for the purposes of weight loss, such as very low-carbohydrate diet, may increase insulin resistance and exacerbate NAFLD even if it can reduce body weight^(9)^. Also, achieving weight loss and maintaining it is often difficult for the most obese patients^(10)^. On the other hand, it has been shown that obese or lean patients with NAFLD benefit more from a healthy diet than from weight reduction^(11)^, suggesting that healthy diet patterns play an important role in the prevention and management of NAFLD. In view of this, several studies have assessed the relationship between these food groups and the likelihood of NAFLD, but results were conflicting^(12–18)^. Thus, to gain a better understanding of the relationship between NAFLD and dietary factors, we searched the literature according to dietary guidelines and guidelines for the prevention and treatment of NAFLD to evaluate the association of the following eleven food groups including refined grains, whole grains, red meat, fish, vegetables, fruits, dairy products, legumes, eggs, nuts and soft drinks, with the likelihood of NAFLD by this meta-analysis^(6,19,20)^.

## Method

This meta-analysis was registered through the International Prospective Register of Systematic Reviews (PROSPERO) as CRD42019120766. This study was reported according to the Meta-analysis Of Observational Studies in Epidemiology (MOOSE) statement and Preferred Reporting Items for Systematic Reviews and Meta-Analyses (PRISMA)^(21,22)^. Similarly, it followed the recommendations of the Cochrane Collaboration Handbook^(23)^. We employed the PICO format (population, intervention, comparison, outcome) to answer the research question: ‘Are food groups (refined grains, whole grains, red meat, fish, vegetables, fruits, dairy products, legumes, eggs, nuts and soft drinks) associated with the occurrence of NAFLD?’. Population: adults with NAFLD; intervention: food groups (refined grains, whole grains, red meat, fish, vegetables, fruits, dairy products, legumes, eggs, nuts and soft drinks); comparison: adults without NAFLD; outcome: the occurrence of NAFLD.

### Search strategy

An electronic search was conducted in the MEDLINE, Embase and Web of Science databases with no restrictions to time, language and publication type. Observational studies addressing the association between food groups and NAFLD were eligible. The following search terms were combined to design the search strategy: ‘Grain’, ‘whole grain’, ‘refined grain’, ‘cereal’, ‘coarse cereal’, ‘meat’, ‘red meat’, ‘white meat’, ‘pork’, ‘beef’, ‘poultry’, ‘domestic fowl’, ‘fish’, ‘diary’, ‘milk’, ‘yogurt’, ‘soy’, ‘legumes’, ‘natto’, ‘tofu’, ‘egg’, ‘vegetable’, ‘fruit’, ‘nut’, soft drink’, ‘carbonate beverage’, ‘carbonated drinks’, ‘sugar beverage’, ‘soda’, ‘nonalcoholic fatty liver disease’, ‘NAFLD’, ‘nonalcoholic steatohepatitis’, ‘liver steatosis’, ‘fatty liver’, ‘hepatic steatosis’, ‘nutritional profile’, ‘nutritional intake’, ‘dietary pattern’, ‘dietary intake’, ‘diet’, ‘nutrition’ and ‘food’. Duplicate publications were removed.

Our two investigators, K. H. and Y. L., independently screened the studies by title, abstract and full text. When the selected studies were identical, agreement was reached; any disagreement was resolved by consulting the third investigator (S. T.). We also manually searched the additional relevant articles from reference lists of retrieved articles.

### Inclusion and exclusion criteria

Inclusion criteria were as follows: (1) participants: adult participants; (2) observational studies: cohort studies, case–control studies or cross-sectional studies that investigated food groups (whole grains, refined grains, vegetables, fruits, soft drinks, fish, red meat, nuts, milk, eggs and legumes) in relation to the likelihood of NAFLD; (3) diagnosis: NAFLD diagnosis that was determined by ultrasound (diffused echogenicity of the liver or increased echogenicity compared with the renal cortex), or by abdominal computed tomography scan (L:S ratio ≤ 1·1, the L:S ratio was calculated from the mean of the liver and spleen measurements) or by multidetector computed tomography scan (a value of the liver:phantom ratio < 30·0) or by MRI (quantified liver fat content) or by proton magnetic resonance spectroscopy (intra-hepatic TAG content more than 5 %) or compatible liver histology^(24–28)^.

Exclusion criteria: (1) animal studies; (2) adolescents or pregnant women; (3) present of hepatitis B surface antigens, antibody against hepatitis C or HIV; (4) excess consumption of alcohol (more than 20 g/d in women or 30 g/d in men) or potentially hepatotoxic drugs (tamoxifen, steroids and amiodarone); (5) other factors which caused hepatic steatosis such as inflammatory bowel disease, coeliac disease or autoimmune hepatitis and (6) diagnosed malignancy.

### Data extraction and risk of bias

Two investigators (K. H. and Y. L.) independently extracted and summarised data from each study. Any discrepancies were resolved by consulting the third investigator (TSH). The quality of the included trials was assessed by the Risk Of Bias In Non-randomized Studies - of Interventions (ROBINS-I) tool^(29)^. It contains seven domains that rank the studies as low, moderate, serious or critical serious. According to the ROBINS-I guidance, if a study is ranked low in all domains, it is considered low risk of bias; if it is ranked low or moderate in all domains, it is considered moderate risk of bias; if it is ranked serious in at least one domain, it is considered serious risk of bias; if it is ranked critical in at least one domain, it is considered critical serious risk of bias^(29)^. All included studies were assessed by two researchers (X. G. and L. Z.), and discrepancies were resolved by consulting the third investigator (S. T.).

### Statistical analysis and data synthesis

We analysed the data using Stata release 15.1 (StataCorp). The results were expressed in terms of OR and 95 % CI. In order to evaluate the weight of each study, the standard error of the logarithmic OR of each study was calculated and taken as the estimated variance of the logarithmic OR. The inverse variance method was adopted^(30)^. Before inclusion in the overall meta-analysis, the results of sex stratification were summarised using the fixed effects model. Different study types (cross-sectional studies or case–control studies or randomised controlled trials or cohort studies) were analysed separately.

Statistical heterogeneity was evaluated by Cochranʼs Q-test and *I* ^2^ statistics, and *P* < 0·1 and *I* ^2^ > 50 % was considered as significant heterogeneity^(31)^. If the heterogeneity was acceptable (*I* ^2^ ≤ 50 %), a fixed effects model was conducted to calculate the pooled OR. Otherwise, a random effects model was adopted. We used ‘metan logor loglb logub, label(namevar=author, yearvar=year) by(study) fixed eform’ command to combine studies without significant heterogeneity (*I* ^2^ ≤ 50 %) and used ‘metan logor loglb logub, label(namevar=author, yearvar=year) by(study) random eform’ command to combine studies with significant heterogeneity (*I* ^2^ > 50). If the number of studies is >5, the causes of heterogeneity were investigated by subgroup analysis based on geographic location (Asia, Europe and America), number of cases (≥1000 *v*. <1000) and dietary assessment method (validated *v*. non-validated). In addition, sensitivity analysis was performed for low-bias studies (if the number of studies > 5). According to the Cochrane Handbook, if ≥10 studies are available, we explore publication bias by using Eggerʼs tests and funnel plots^(32,33)^.

## Results

A total of 7892 potentially relevant articles were identified through literature searching in MEDLINE, Embase and Web of Science, and 2322 duplicate articles were excluded. The remaining 5570 articles undergone a title and abstract screening, and 5499 were further excluded as they did not fulfil the inclusion criteria.

**Fig. 1.**
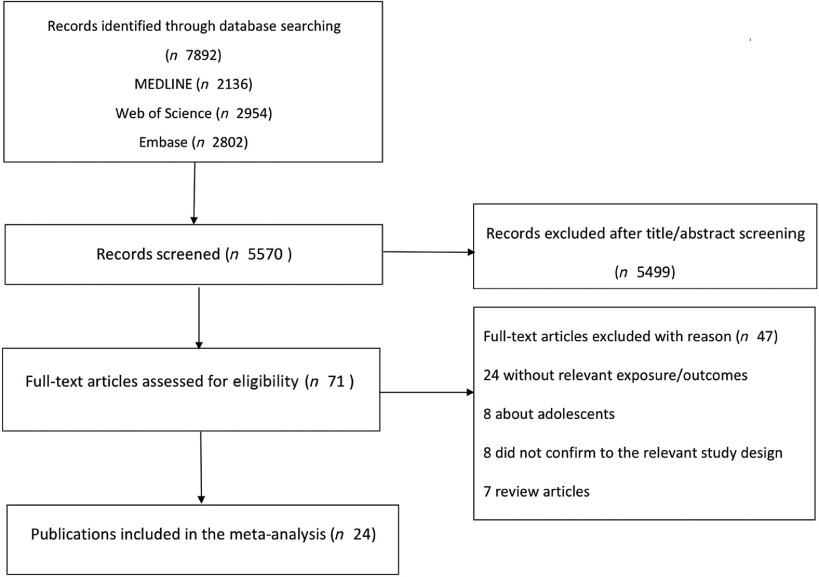
Flow diagram of literature search and study selection.

In total, seventy-one articles remained for full-text evaluation. Among them, forty-seven articles were excluded because seven were review articles, eight were about adolescents, twenty-four were without the relevant exposure/outcomes and eight did not conform to the relevant study design. Finally, twenty-four articles were identified and included in this systematic review and meta-analysis^(12–15,18,34–52)^. The study selection process is described in Fig. 1. Among them, fifteen were cross-sectional studies and nine were case–control studies. Of the studies, seventeen studies were conducted in Asia (nine in China, one in South Korea, two in Iran, three in Israel and two in Japan), five in European (three in Greece, two in Italy) and two in America. The main characteristics of the included studies are illustrated in online Supplementary Tables S1 and S2.

### Quality assessment

Fifteen studies were evaluated to have a low risk of bias, and nine studies had a moderate risk of bias. The quality of included studies ranged from low to moderate risk of bias as shown in online Supplementary Table S3. Bias risk of each domain of the included studies is also shown in online Supplementary Table S3.

### Red meat

**Fig. 2.**
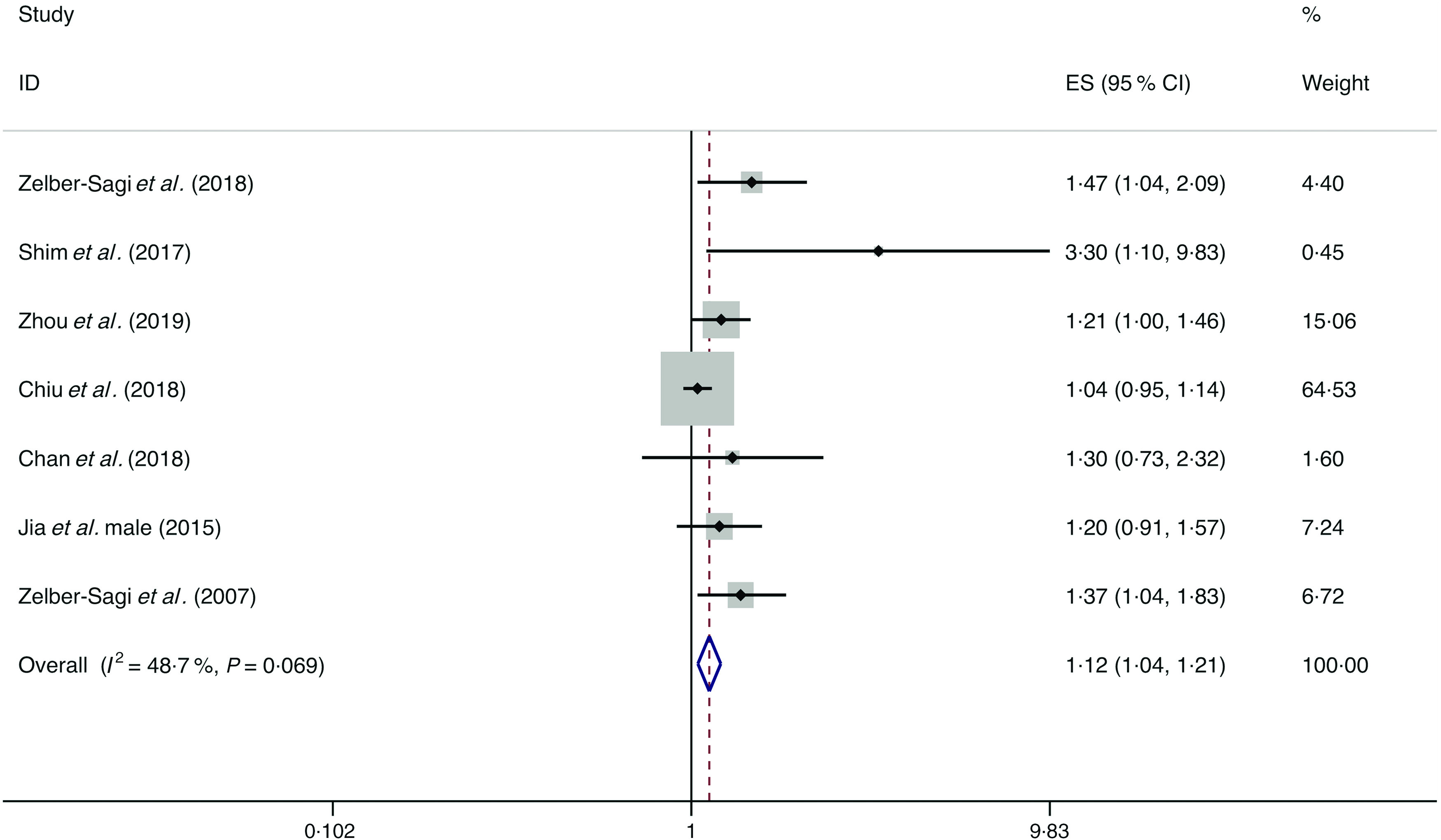
Fixed effects meta-analysis of cross-sectional studies that examined red meat consumption and non-alcoholic fatty liver disease (NAFLD) risk. Weights are from fixed effects analysis. ES, effect size.

A total of eight studies assessed the effect of red meat consumption on the likelihood of NAFLD, which include seven cross-sectional studies (with 5141 cases) and one case–control study (with 2974 cases)^(18,34,36,38,44,48,49,52)^. Meta-analysis results from the seven homogeneous cross-sectional studies (*I* ^2^ = 48·7 %, *P* = 0·069) showed a positive association between red meat consumption and the likelihood of NAFLD (OR = 1·121; 95 % CI 1·042, 1·207; *P* = 0·002) (Fig. 2). Also, the result from the case–control study found a positive association (OR = 1·150; 95 % CI 1·023, 1·293; *P* = 0·020).

Further, subgroup analyses were conducted by risk of bias, geographic location, number of cases and dietary assessment. Except for the two studies with ≥1000 cases by which the pooled result showed no association of red meat consumption with the likelihood of NAFLD (*I* ^2^ = 0·0 %, *P*_heterogeneity_ = 0·340; OR = 1·005; 95 % CI 0·967, 1·150; *P* = 0·227), the results still showed positive associations between red meat consumption and the likelihood of NAFLD in the studies with low risk of bias and <1000 cases, and in the studies from the analyses by geographic location and dietary assessment. Evidence of significant heterogeneity in subgroup analysis was only found in the studies with validated dietary assessment (*I* ^2^ = 56·4 %) (online Supplementary Table S4).

### Soft drinks

**Fig. 3.**
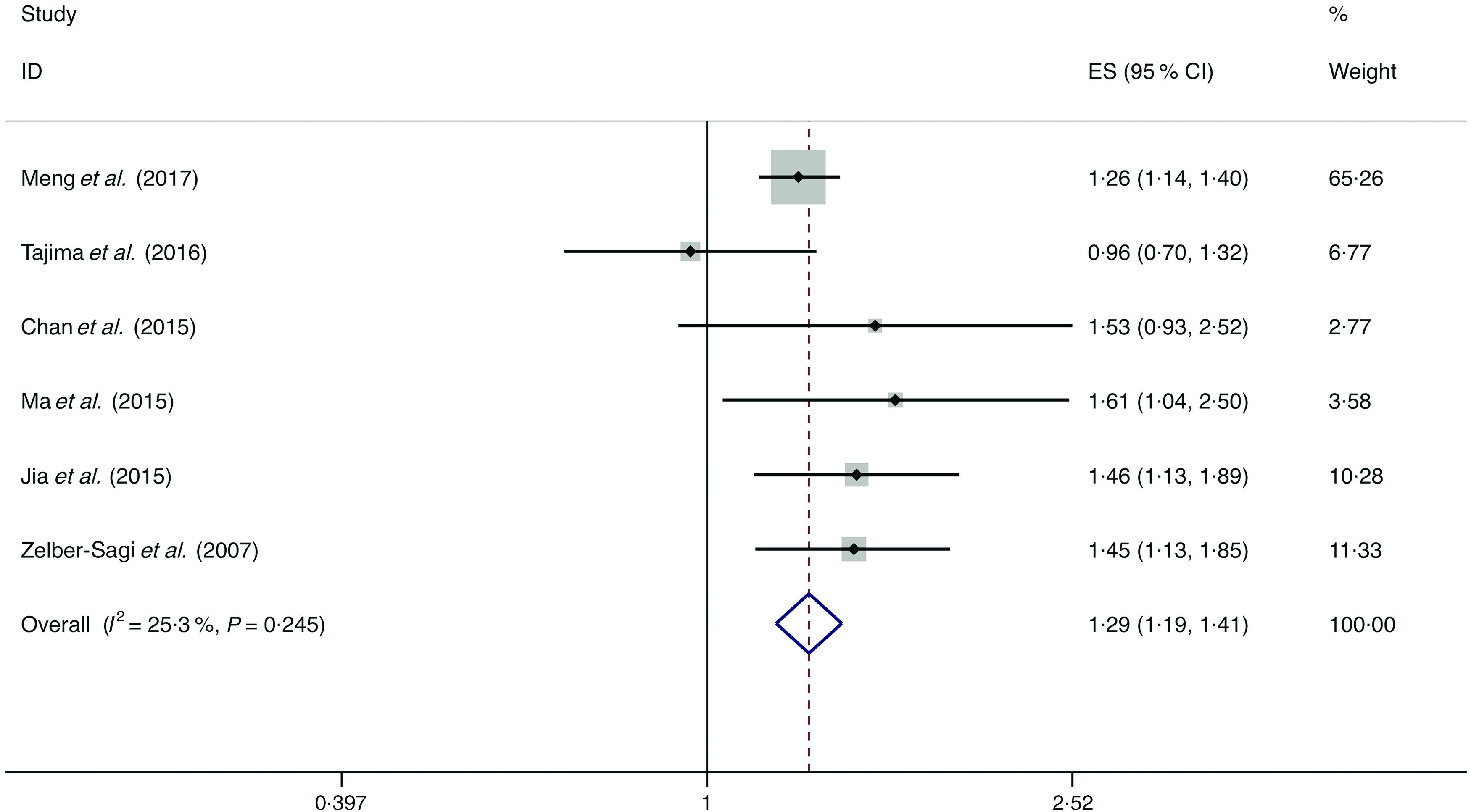
Fixed effects meta-analysis of cross-sectional studies that examined soft drink consumption and non-alcoholic fatty liver disease (NAFLD) risk. Weights are from fixed effects analysis. ES, effect size.

A total of seven studies assessed the effect of soft drink consumption on the likelihood of NAFLD, which include six cross-sectional studies (with 9887 cases) and one case–control study (with sixty cases)^(12,34,38,40,41,46,49)^. Meta-analysis results from the six homogeneous cross-sectional studies (*I* ^2^ = 25·3 %, *P* = 0·245) showed that soft drink consumption was positively correlated with the likelihood of NAFLD (OR = 1·294; 95 % CI 0·191, 1·406; *P* = 0·000) (Fig. 3). However, the result from the case–control study found no association between soft drink intake and the possibility of NAFLD (OR = 2·000; 95 % CI 0·894, 4·472; *P* = 0·091).

Stratified by risk of bias, geographic location, number of cases and dietary assessment in subgroup analyses, the results still indicated positive correlations between beverage intake and the likelihood of NAFLD. Evidence of no significant heterogeneity was found in subgroup analysis (online Supplementary Table S5).

### Nuts

**Fig. 4.**
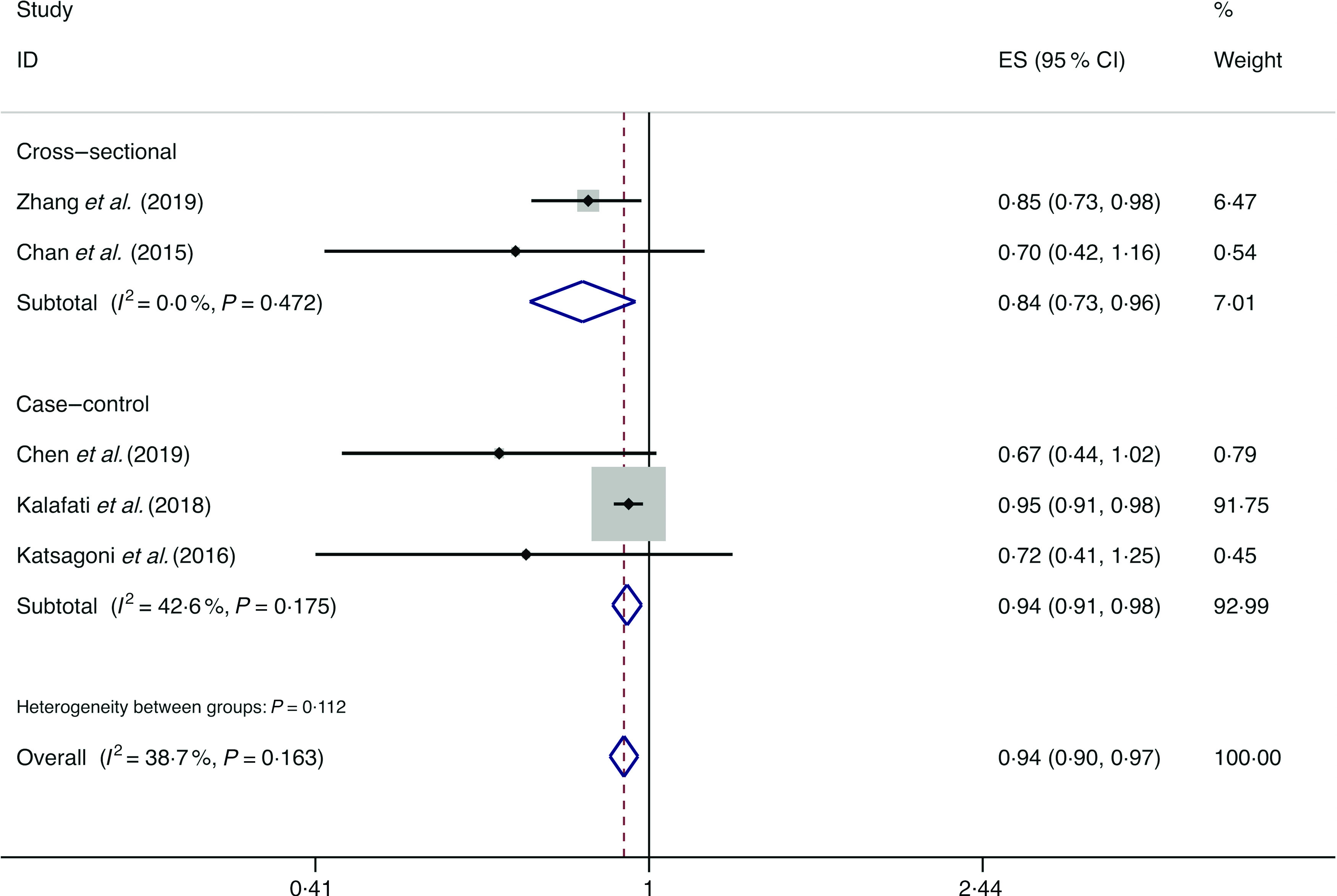
Fixed effects meta-analysis of prospective studies that examined nut consumption and non-alcoholic fatty liver disease (NAFLD) risk. Weights are from fixed effects analysis. ES, effect size.

A total of five studies assessed the effect of nut consumption on the likelihood of NAFLD, which include two cross-sectional studies (with 4737 cases) and three case–control studies (with 768 cases)^(15,34,35,39,51)^. A negative association of nut intake with the possibility of NAFLD was observed among the cross-sectional studies (*I* ^2^ = 0·0 %, *P*_heterogeneity_ = 0·472; OR = 0·837; 95 % CI 0·727, 0·965; *P* = 0·014) and case–control studies (*I* ^2^ = 42·6 %, *P*_heterogeneity_ = 0·175; OR = 0·943, 95 % CI 0·907, 0·980; *P* = 0·003) (Fig. 4).

### Whole grains

**Fig. 5.**
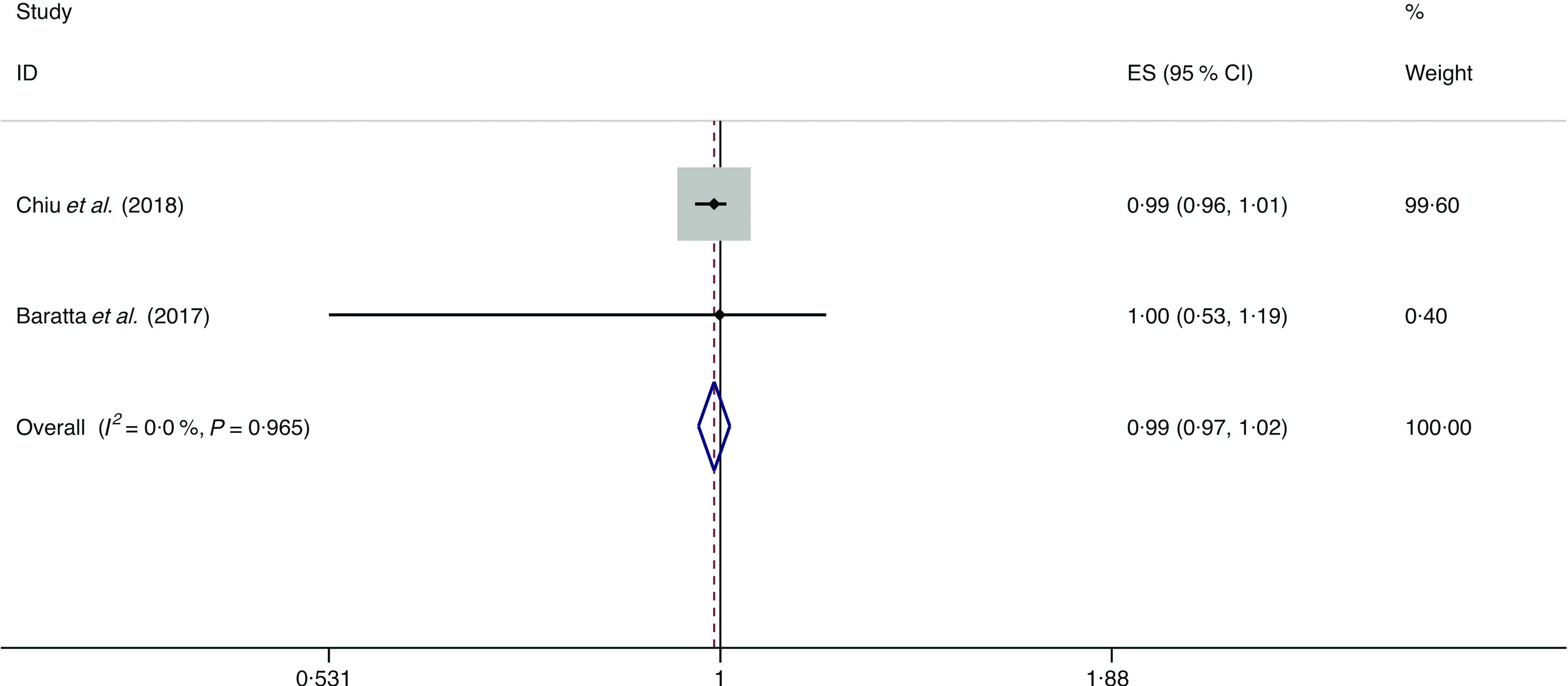
Fixed effects meta-analysis of cross-sectional studies that examined nut consumption and non-alcoholic fatty liver disease (NAFLD) risk. Weights are from fixed effects analysis. ES, effect size.

A total of three studies assessed the effect of whole-grain consumption on the likelihood of NAFLD, which include two cross-sectional studies (with 2394 cases) and one case–control study (with seventy-three cases)^(14,36,37)^. No significant association between whole-grain consumption and the likelihood of NAFLD was observed among the cross-sectional studies (*I* ^2^ = 0·0 %, *P*_heterogeneity_ = 0·965; OR = 0·990; 95 % CI 0·965, 1·015; *P* = 0·439) and case–control study (OR = 1·029; 95 % CI 0·993, 1·067; *P* = 0·119) (Fig. 5).

### Refined grains

**Fig. 6.**
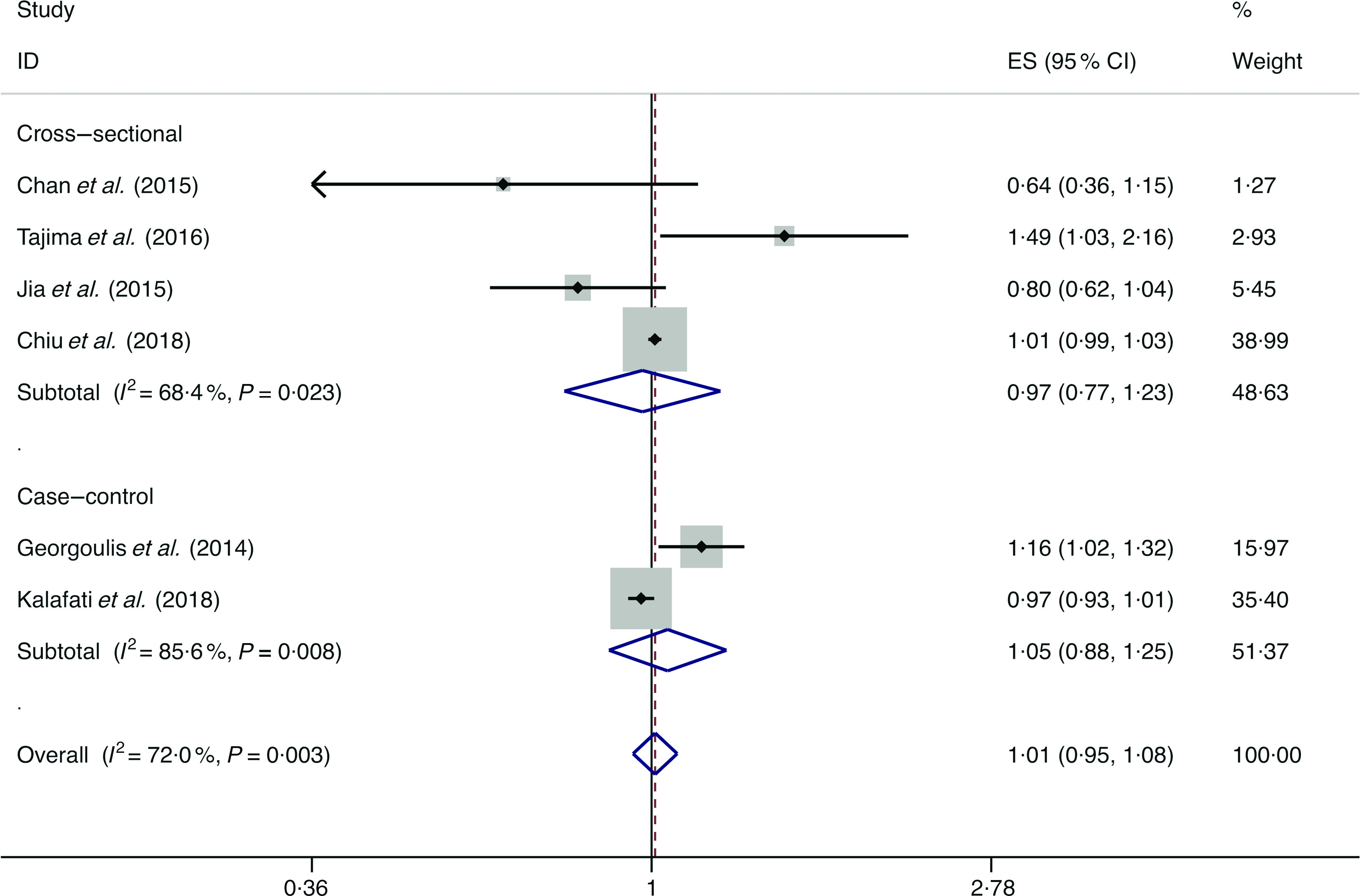
Random effects meta-analysis of prospective studies that examined refined grain consumption and non-alcoholic fatty liver disease (NAFLD) risk. Weights are from random effects analysis. ES, effect size.

A total of six studies assessed the effect of refined grain consumption on the likelihood of NAFLD, which include four cross-sectional studies (with 3509 cases) and two case–control studies (with 207 cases)^(34,36–39,46)^. No significant association between refined grain consumption and the likelihood of NAFLD was observed among the cross-sectional studies (*I* ^2^ = 68·4 %, *P*_heterogeneity_ = 0·023; OR = 0·973; 95 % CI 0·769, 1·230; *P* = 0·818) and case–control study (*I* ^2^ = 85·6 %, *P*_heterogeneity_ = 0·008; OR = 1·050; 95 % CI 0·880, 1·253; *P* = 0·591) (Fig. 6).

### Fish

**Fig. 7.**
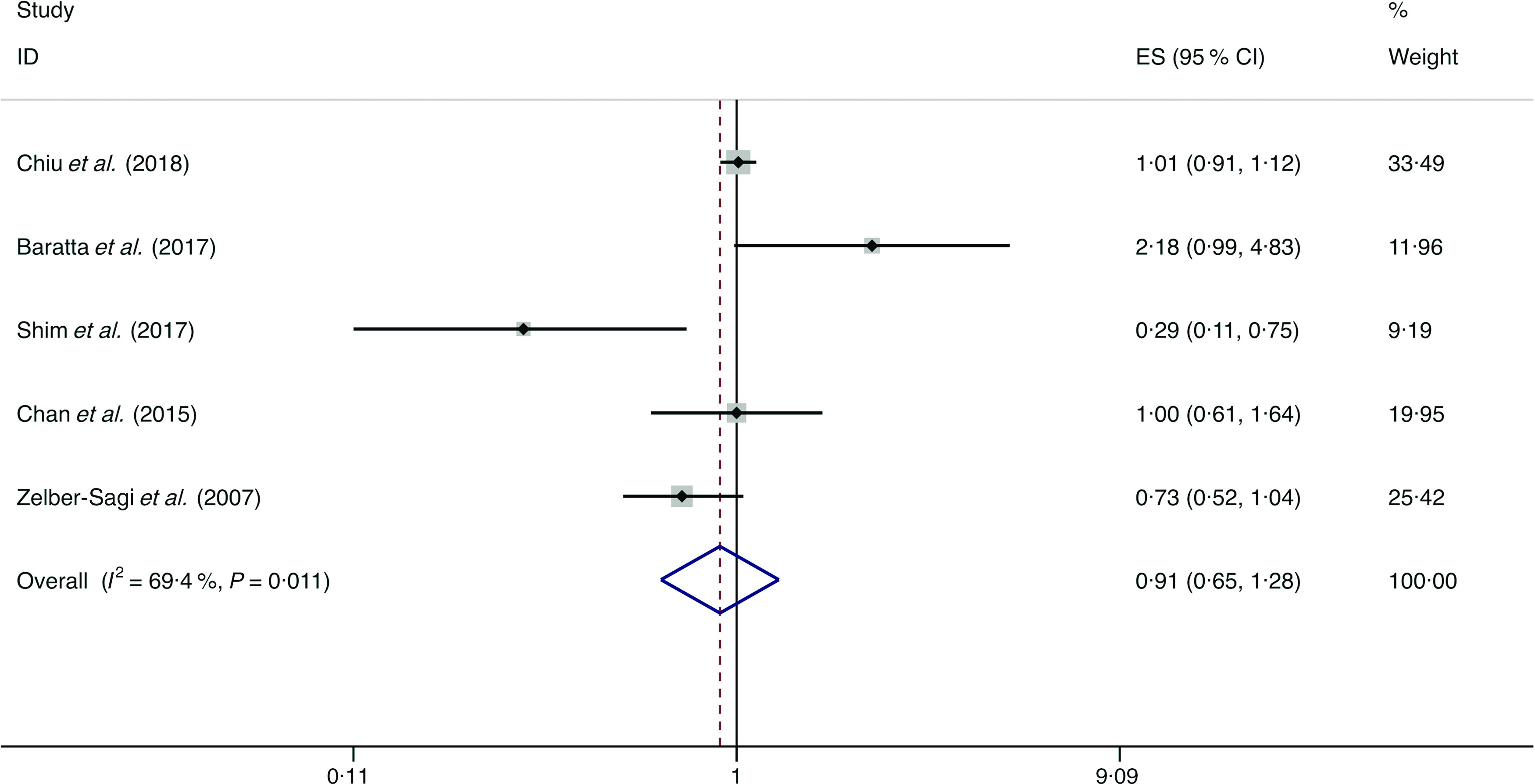
Random effects meta-analysis of cross-sectional studies that examined fish consumption and non-alcoholic fatty liver disease (NAFLD) risk. Weights are from random effects analysis. ES, effect size.

A total of six studies assessed the effect of fish consumption on the likelihood of NAFLD, which include five cross-sectional studies (with 2780 cases) and one case–control study (with 134 cases)^(14,18,34,36,39,49)^. Meta-analysis results from the five heterogeneous cross-sectional studies (*I* ^2^ = 69·4 %, *P* = 0·011) showed no significant association between fish consumption and the likelihood of NAFLD (OR = 0·908; 95 % CI 0·647, 1·276; *P* = 0·579) (Fig. 7). However, the result from the case–control study showed fish consumption was negatively associated with the possibility of NAFLD (OR = 0·845; 95 % CI 0·751, 0·950; *P* = 0·005).

### Fruits

**Fig. 8.**
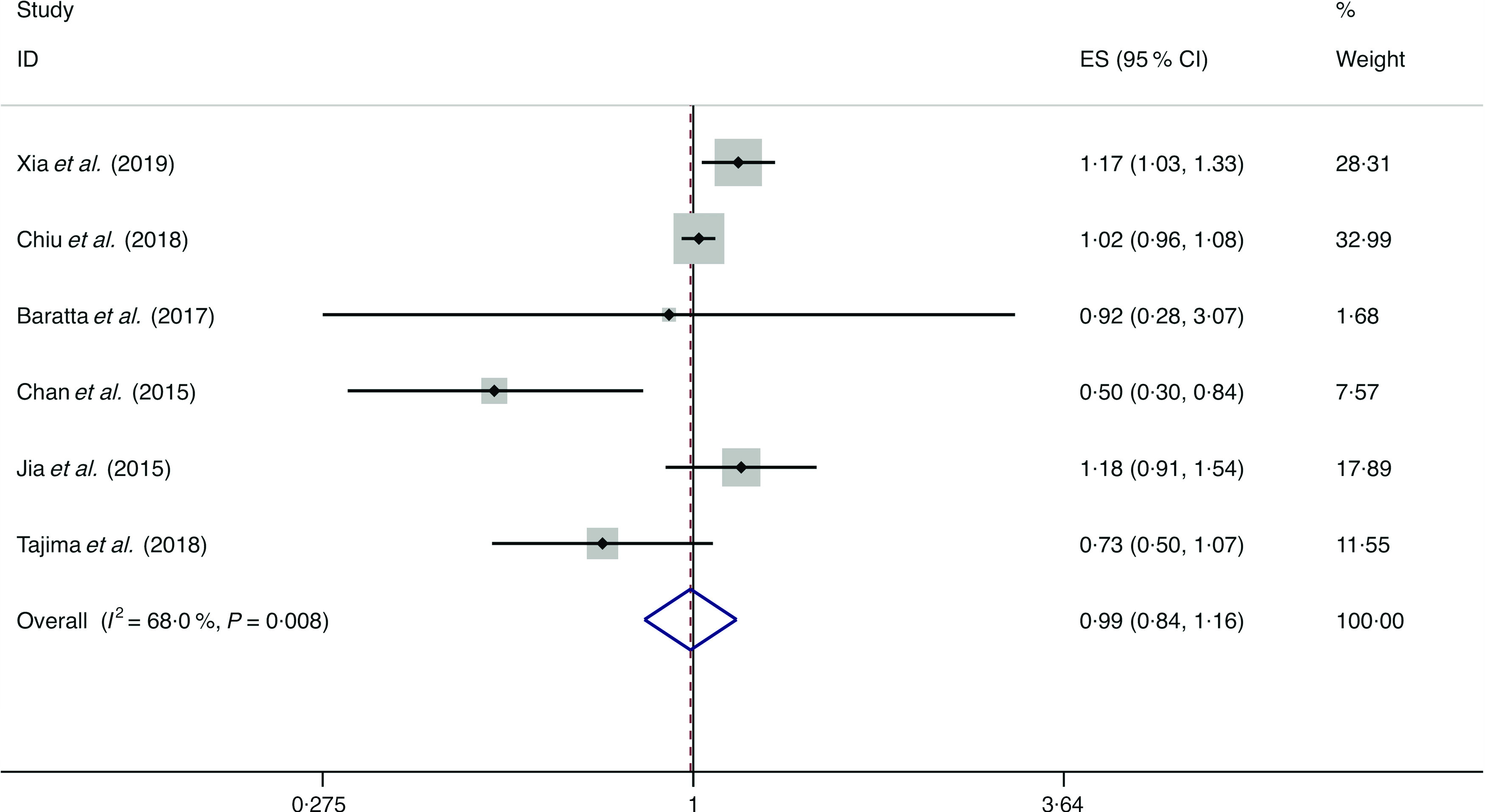
Random effects meta-analysis of cross-sectional studies that examined fruit consumption and non-alcoholic fatty liver disease (NAFLD) risk. Weights are from random effects analysis. ES, effect size.

**Fig. 9.**
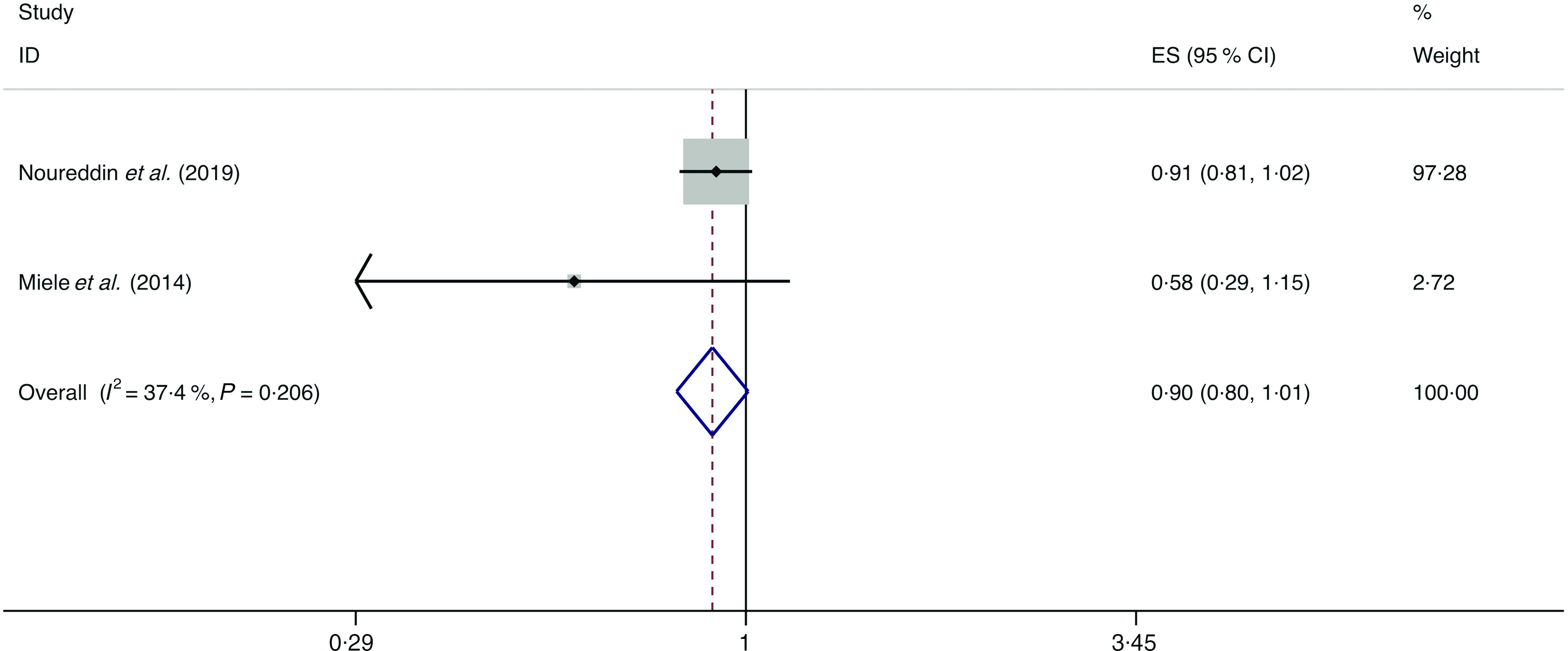
Fixed effects meta-analysis of case–control studies that examined fruit consumption and non-alcoholic fatty liver disease (NAFLD) risk. Weights are from fixed effects analysis. ES, effect size.

A total of eight studies assessed the effect of fruit consumption on the likelihood of NAFLD, which include six cross-sectional studies (with 11 861 cases) and two case–control studies (with 2168 cases)^(14,34,36,38,42,44,45,47)^. There was no significant association between fruit intake and the likelihood of NAFLD among the cross-sectional studies (*I* ^2^ = 68·0 %, *P*_heterogeneity_ = 0·008; OR = 0·991; 95 % CI 0·844, 1·163; *P* = 0·907) (Fig. 8) and case–control studies (*I* ^2^ = 37·4 %, *P*_heterogeneity_ = 0·206; OR = 0·899; 95 % CI 0·802, 1·007; *P* = 0·066) (Fig. 9).

In subgroup analysis, the pooled result from the studies with <1000 cases showed a negative correlation between fruit intake and the likelihood of NAFLD (*I* ^2^ = 0·0 %, *P*_heterogeneity_ = 0·437; OR = 0·651; 95 % CI 0·483, 0·878; *P* = 0·005), whereas the results of all other subgroup analyses were consistent with the above overall analysis. Evidence of significant heterogeneity was still observed in the stratified analyses of the Asian studies (*I* ^2^ = 74·4 %), the studies with ≥1000 cases (*I* ^2^ = 55·2 %) and the studies with validated dietary assessment (*I* ^2^ = 72·2 %) (online Supplementary Table S6).

### Vegetables

**Fig. 10.**
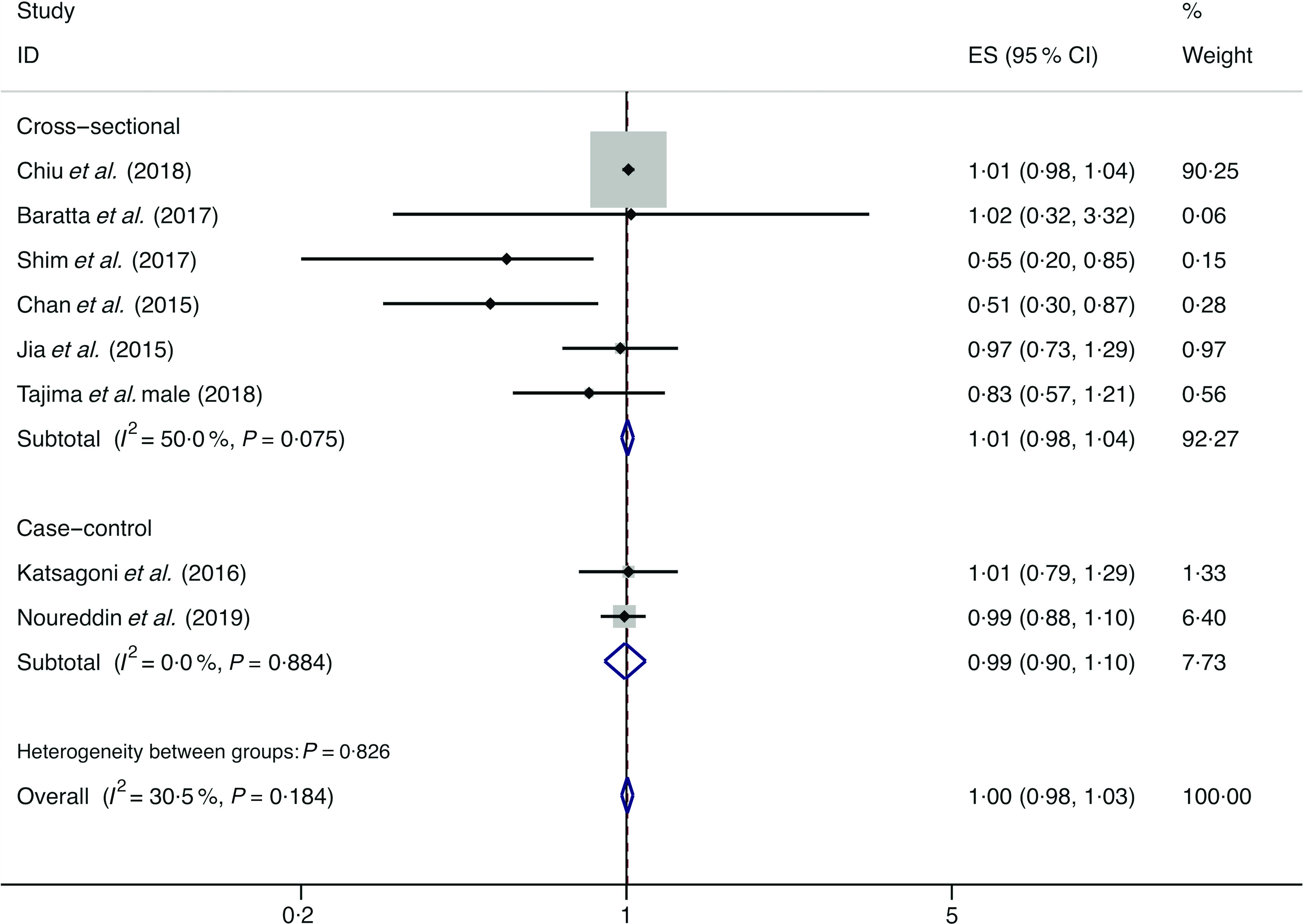
Fixed effects meta-analysis of prospective studies that examined vegetable consumption and non-alcoholic fatty liver disease (NAFLD) risk. Weights are from fixed effects analysis. ES, effect size.

A total of eight studies assessed the effect of vegetable consumption on the likelihood of NAFLD, which include six cross-sectional studies (with 4523 cases) and two case–control studies (with 3074 cases)^(14,15,34,36,38,44,45,47)^. There was no significant association between vegetable intake and the likelihood of NAFLD among the cross-sectional studies (*I* ^2^ = 50·0 %, *P*_heterogeneity_ = 0·075; OR = 1·005; 95 % CI 0·976, 1·035; *P* = 0·725) and case–control studies (*I* ^2^ = 0·0 %, *P*_heterogeneity_ = 0·884; OR = 0·993; 95 % CI 0·897, 1·1000; *P* = 0·898) (Fig. 10).

In subgroup analysis, the pooled result from the studies with <1000 cases showed a negative correlation between vegetables intake and the likelihood of NAFLD (*I* ^2^ = 0·0 %, *P*_heterogeneity_ = 0·398; OR = 0·696; 95 % CI 0·528, 0·916; *P* = 0·010), whereas the results of all other subgroup analyses were consistent with the above overall analysis. Evidence of significant heterogeneity was still found in the stratified analyses of the Asian studies (*I* ^2^ = 60·0 %) and the studies with validated dietary assessment (*I* ^2^ = 59·8 %) (online Supplementary Table S7).

### Eggs

**Fig. 11.**
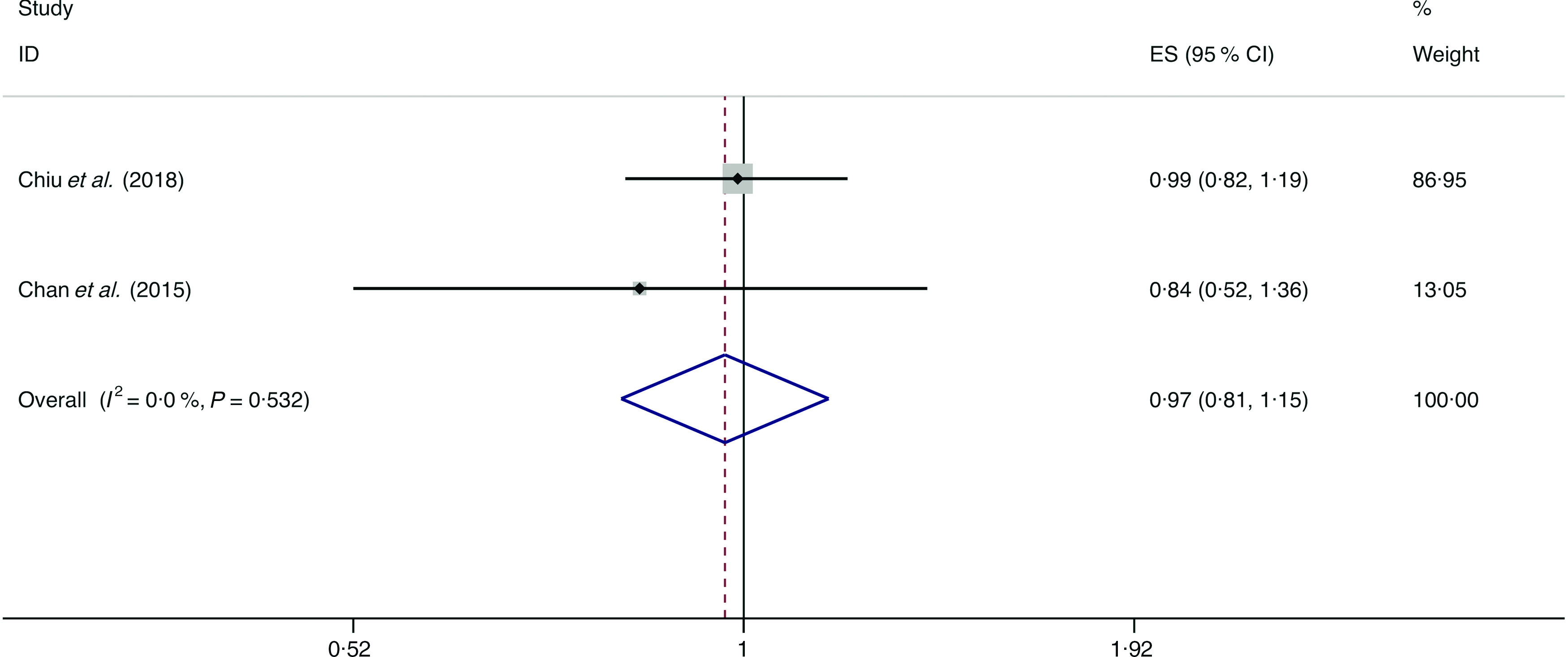
Fixed effects meta-analysis of cross-sectional studies that examined egg consumption and non-alcoholic fatty liver disease (NAFLD) risk. Weights are from fixed effects analysis. ES, effect size.

A total of three studies assessed the effect of egg consumption on the likelihood of NAFLD, which include two cross-sectional studies (with 2131 cases) and one case–control study (with 169 cases)^(34,36,43)^. Neither the cross-sectional studies (*I* ^2^ = 0·0 %, *P*_heterogeneity_ = 0·532; OR = 0·969; 95 % CI 0·815, 1·153; *P* = 0·722) nor the case–control study (OR = 0·966; 95 % CI 0·453, 2·060; *P* = 0·929) showed the association between egg consumption and NAFLD (Fig. 11).

### Dairy products

**Fig. 12.**
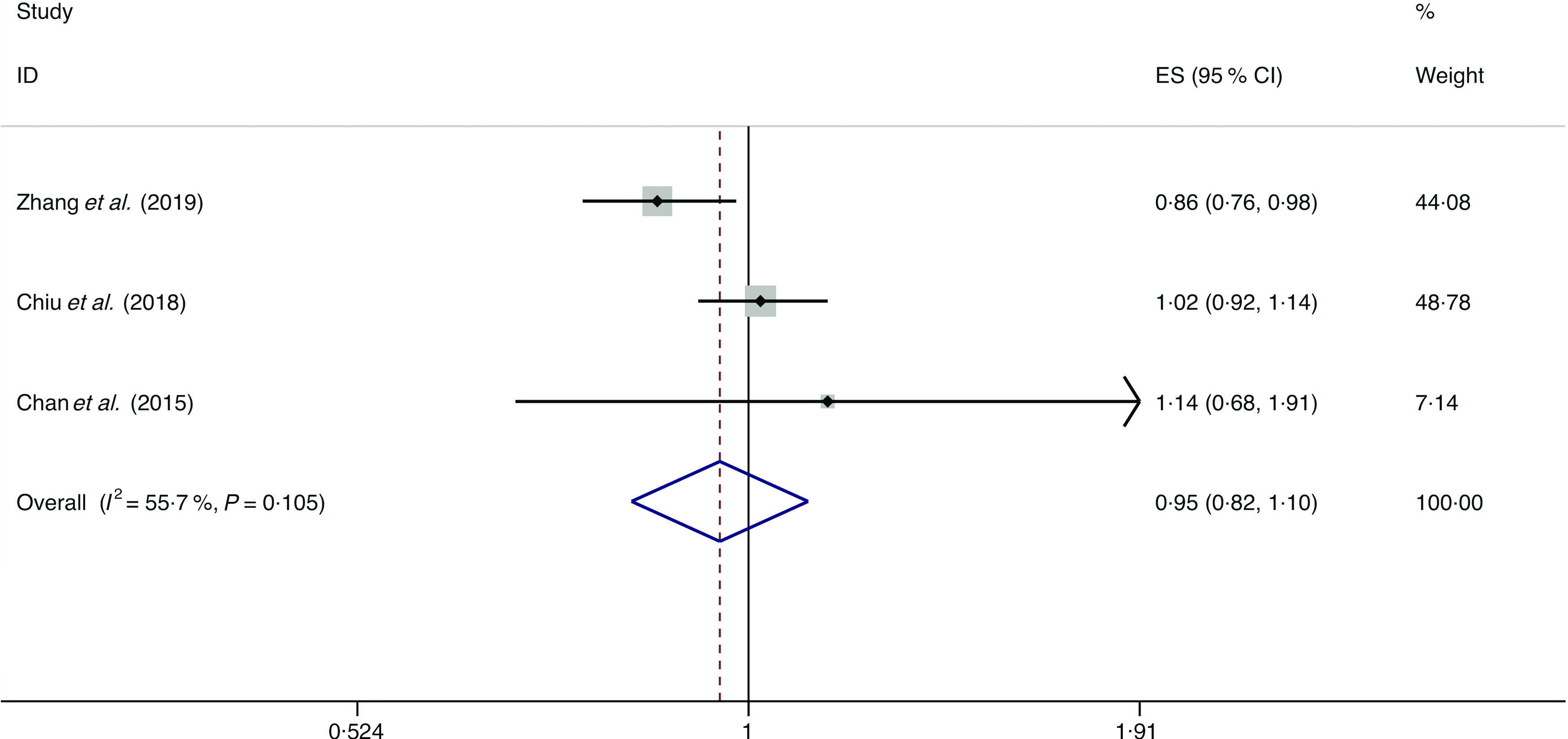
Random effects meta-analysis of cross-sectional studies that examined dairy product consumption and non-alcoholic fatty liver disease (NAFLD) risk. Weights are from random effects analysis. ES, effect size.

A total of four studies assessed the effect of dairy product consumption on the likelihood of NAFLD, which include three cross-sectional studies (with 6789 cases) and one case–control study (with 143 cases)^(34,36,39,50)^. Meta-analysis results from the three heterogeneous cross-sectional studies (*I* ^2^ = 55·7 %, *P* = 0·105) showed no significant association between dairy product consumption and the likelihood of NAFLD (OR = 0·954; 95 % CI 0·824, 1·104; *P* = 0·524) (Fig. 12). However, the result from the case–control study showed that dairy product consumption was positively associated with the possibility of NAFLD (OR = 1·192; 95 % CI 1·002, 1·419; *P* = 0·048).

### Legumes

**Fig. 13.**
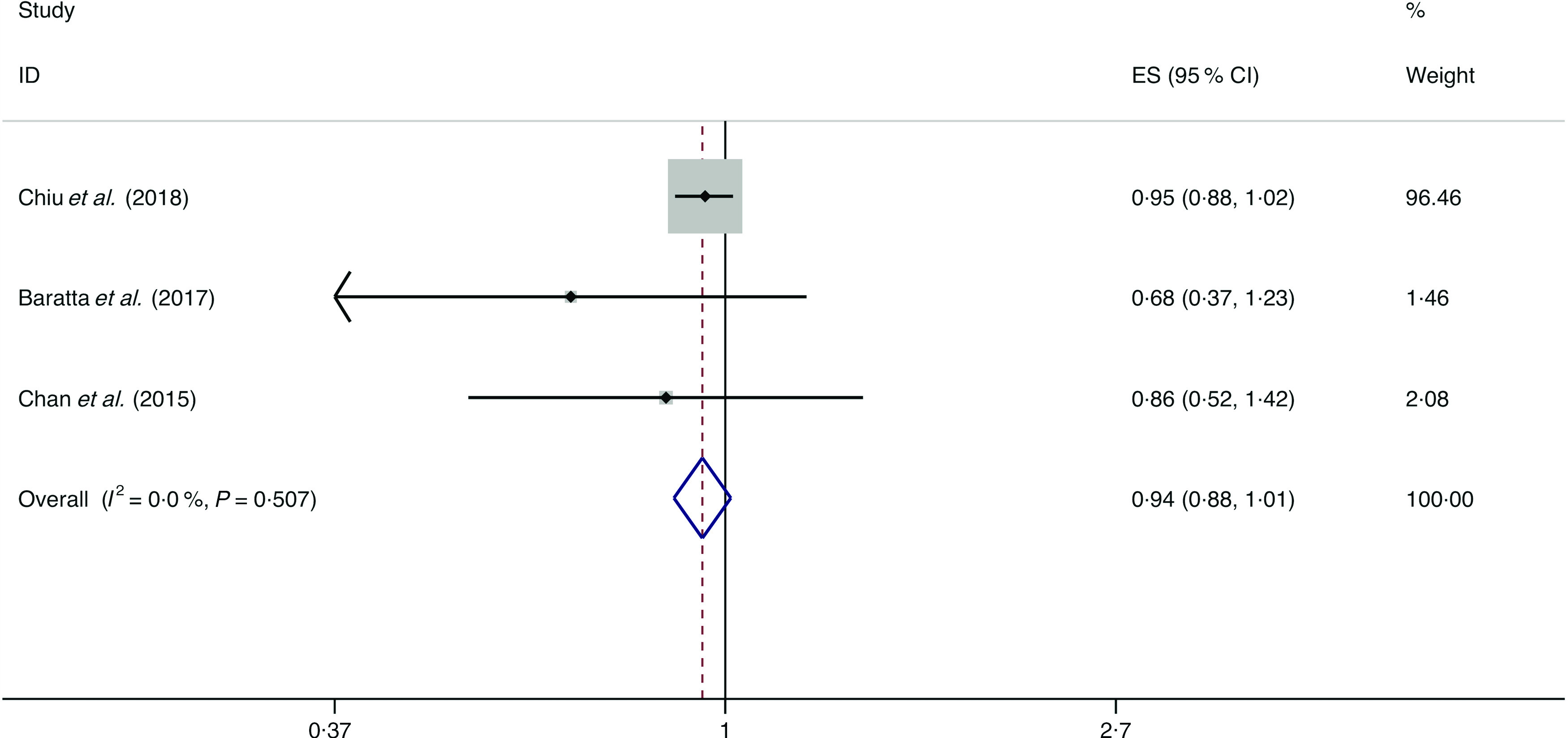
Random effects meta-analysis of cross-sectional studies that examined legume consumption and non-alcoholic fatty liver disease (NAFLD) risk. Weights are from random effects analysis. ES, effect size.

A total of four studies assessed the effect of legume consumption on the likelihood of NAFLD, which include three cross-sectional studies (with 2614 cases) and one case–control study (with 196 cases)^(13,14,34,36)^. Meta-analysis results from the three homogeneous cross-sectional studies (*I* ^2^ = 0·0 %, *P* = 0·507) showed no significant association between legume consumption and the likelihood of NAFLD (OR = 0·943; 95 % CI 0·877, 1·014; *P* = 0·115) (Fig. 13). However, the result from the case–control study showed legume consumption was negatively associated with the possibility of NAFLD (OR = 0·730; 95 % CI 0·637, 0·836; *P* = 0·000).

### Sensitivity analyses

In the sensitivity analyses, the findings from the studies with low risk of bias suggest a stronger positive association between red meat (*I* ^2^ = 0·0 %, *P*_heterogeneity_ = 0·817; OR = 1·218; 95 % CI 1·018, 1·458; *P* = 0·031) and soft drinks (*I* ^2^ = 0·0 %, *P*_heterogeneity_ = 0·880; OR = 1·575; 95 % CI 1·133, 2·189; *P* = 0·007) intake and the possibility of NAFLD (online Supplementary Tables S4 and S5). Moreover, an inverse association was found by sensitivity analysis between vegetable consumption and NAFLD (*I* ^2^ = 10·2 %, *P*_heterogeneity_ = 0·291; OR = 0·574; 95 % CI 0·353, 0·932; *P* = 0·025) in the studies with low risk of bias (online Supplementary Table S7).

## Discussion

In this meta-analysis, the results revealed that intake of red meat and soft drinks was associated with an increase in the likelihood of NAFLD, whereas intake of nuts was negatively associated with the possibility of NAFLD. It is noteworthy that most foods included in the meta-analysis (whole grains, refined grains, fish, fruits, vegetables, eggs, dairy products and legumes) may have no significant effect on the likelihood of NAFLD.

To our knowledge, this is the first meta-analysis investigating the relationship between food groups (refined grains, whole grains, fish, red meat, vegetables, fruits, soft drinks, eggs, legumes, nuts and dairy products) and the likelihood of NAFLD. The pooled results of our meta-analysis are in accordance with other systematic reviews and meta-analyses, indicating that consumption of red meat and sugar- and artificially sweetened soda is positively associated with NAFLD^(53,54)^. Firstly, red meat rich in saturated fat increases hepatic lipid accumulation and insulin resistance via reducing lipid oxidation and increasing lipid synthesis^(55,56)^. Additionally, haem Fe intake reduces insulin sensitivity through cellular oxidation stress^(57)^. Red meat is often processed with a lot of Na and preserved with nitrites, which is related to increase the likelihood of insulin resistance and NAFLD^(58,59)^. Secondly, several studies have also shown that higher consumption of soft drinks is associated with a greater likelihood of NAFLD and a series of metabolic syndromes^(60,61)^. Soft drinks provide a large amount of sugar and excessive energy content, which led to rapidly increased insulin level and postprandial glucose^(60,62)^. Lebda *et al.* stated that long-term intake of soft drinks is prospectively associated with the level of ALT which represents liver inflammation^(63)^.

Consistent with other studies, our meta-analysis shows that nut consumption reduces the likelihood of NAFLD. A study with 12 946 participants indicated that nut consumption was positively associated with healthier nutrition and lifestyle^(64)^; another study displayed that risk factors associated with NAFLD and CVD were improved after regular nut consumption^(65)^.

However, the current research still fails to reach a consistent conclusion on the possibility of NAFLD with intake of fish, legumes, fruits, vegetables and dairy products. In the cross-sectional studies, the pooled results showed that no significant associations were observed between consumption of both fish (OR = 0·908) and legumes (OR = 0·943) and the likelihood of NAFLD; but in the case–control studies, the results showed that consumption of both fish (OR = 0·845) and legumes (OR = 0·730) decreased the possibility of NAFLD. In the cross-sectional studies and the case–control studies, the pooled results showed no significant associations between intake of both fruits and vegetables and the likelihood of NAFLD; but in the cross-sectional studies with <1000 cases, the pooled results showed that consumption of both fruits (OR = 0·651) and vegetables (OR = 0·696) significantly reduced the possibility of NAFLD. Moreover, in the cross-sectional studies with low risk of bias, the pooled results showed that intake of vegetables (OR = 0·574) also reduced the possibility of NAFLD. On the contrary, in the cross-sectional studies, the pooled results showed that no significant association was observed between dairy product consumption and the likelihood of NAFLD, but in the case–control study, the result showed that dairy product consumption increased the possibility of NAFLD (OR = 1·192)^(50)^. Taken together, the above findings suggest that higher consumption of fish, legumes, fruits and vegetables appeared to have a protective trend against NAFLD likelihood, while intake of more milk may be an adverse effect on the likelihood of NAFLD.

*n*-3 PUFA in fish and isoflavones in legumes have been shown to reduce lipid accumulation and liver enzyme levels, to improve insulin sensitivity and to have anti-inflammatory effects and thus are associated with the prevention of the development of hepatic steatosis, NAFLD, non-alcoholic steatohepatitis and fibrosis^(66–68)^. Fruits and vegetables are rich in fibre, antioxidants such as polyphenols, which help prevent the occurrence of NAFLD. In addition to the antioxidant effect, polyphenols also have beneficial effects on metabolic homoeostasis *in vivo* and *in vitro* NAFLD model, with anti-inflammatory and anti-fibrosis effects. In general, they inhibit *de novo* fat synthesis and stimulate *β*-oxidation in the liver^(69)^. On the other hand, the case–control study^(50)^ as mentioned above found that dairy products (mainly referring to cheese) increased the likelihood of NAFLD. This may be related to the fact that cheese contains more SFA that increase liver steatosis^(39)^. More studies are needed to further confirm the relationship between fish, legumes, fruits, vegetables and dairy products and the likelihood of NAFLD.

In recent years, on the basis of the components of the Mediterranean diet, the literature reports its beneficial effects in preventing major chronic diseases, including obesity, diabetes, CVD and some forms of cancers^(70–73)^. More importantly, a growing body of evidence has supported the idea that the Mediterranean diet, associated with exercise and cognitive behaviour therapy, may be the reference nutritional profile for the prevention and the treatment of NAFLD patients^(74–76)^. It is characterised by an abundance of consumption of whole grains, vegetables, fruits, legumes, nuts and olive oil (rich in monounsaturated fat); a moderate intake of fish and poultry; low consumption of red/processed meat and dairy products and low to moderate consumption of alcohol during meals^(75)^. From our meta-analysis results, a high to moderate intake of nuts, legumes, fish, eggs, whole grains and vegetables, and low consumption of red meat and soft drinks should be recommended for patients with NAFLD, which is roughly similar to the diet composition of the Mediterranean diet. At present, the Mediterranean diet is the latest recommended dietary pattern for NAFLD patients in EASL-EASD-EASO clinical practice guidelines^(6)^.

Most of the studies measured food intake with a FFQ, which is easy for the administration to assess long-term habitual food consumption. It contains numerous food items with specific serving sizes and frequency categories and has the following advantages: first, since the questionnaire is structured, it is convenient and simple; second, the implementation is simple and easy to understand, and the participation rate of subjects is high; third, it can be used to analyse the correlation between specific nutrients or related foods and diseases. However, there are some limitations. First, it may cause bias due to errors in the estimation of portion sizes; second, cooking methods and seasoning dosage are not easy to estimate; there may be a large error range. Although the debate about the utility of FFQ in nutritional epidemiological studies is often polarised, dietary data derived from it have proven useful in addressing important research questions^(77)^. Of the twenty-four studies included in our meta-analysis, twenty-one studies used FFQ to investigate dietary intake. Since the implementation is simple and the content design is easy to understand, subjects will be more willing to cooperate, thus guaranteeing the reliability of the results of this meta-analysis.

Some strengths of this meta-analysis are as follow: (1) we investigated a variety of foods; (2) all the studies included in our meta-analysis were low to moderate bias risk; (3) all the studies included in our meta-analysis used multiple logistic regressions to reduce the effect of confounders on the correlation of NAFLD with food consumption. However, several limitations of this meta-analysis should be noted. First, because the overall number of studies included was small, subgroup and sensitivity analyses were limited to four of the eleven food groups (red meat, soft drinks, fruits and vegetables). Consistent with this, publication bias could not be tested as the overall low number of included studies. Also, the results of several food groups may be biased due to too few studies included (eggs = three studies, dairy products = four studies, legumes = four studies, whole grains = three studies). Lastly, since most studies do not stratify food intake, it is not possible to perform linear or nonlinear dose–response of different food groups. Also, the included studies used different units or different standards to measure food groups.

In summary, this meta-analysis with twenty-four studies identified eleven food groups associating the likelihood of NAFLD. The results were broadly consistent with the current dietary recommendation for the management of NAFLD. Larger and more precise studies are required to further assess the association and the underlying mechanisms between food groups and the possibility of NAFLD.

## Supplementary material

For supplementary material accompanying this paper visit http://dx.doi.org/10.1017/S0007114520000914. click here to view supplementary material
