# Supplementary material for: Food groups and the likelihood of non-alcoholic fatty liver disease: a systematic review and meta-analysis
Source: Br J Nutr. 2020 Mar 6;124(1):1–13. doi: 10.1017/S0007114520000914 (PMC7512147; doi:10.1017/S0007114520000914)
Supplement: Supplementary file 1 [file S0007114520000914sup001.docx]

**Online Supplementary Material**

**Supplementary Table 1** Characteristics of cross-sectional studies included in the meta-analysis^1^

| Author | Year | Location | Participant | Diagnosed method of NAFLD | Dietary measurement | Food groups | Confounder adjusted |
| --- | --- | --- | --- | --- | --- | --- | --- |
| Zhang et al.(a) | 2019 | China | NAFLD: n=4658, age=42, 71.6%M  Control: n=19731, age=41.2, 41.2%M | Validated FFQ | Abdominal ultrasound | Dairy | Age, sex, BMI, smoking stuatus, alcohol drinking status, education level, working status, household income, physical activity, family history of disease, total energy intake, carbohydrate intake, total fat intake, EPA + DHA intake, soft drinks intake, vegetables intake, fruits intake, sweet foods intake, milk intake, hypertension, diabetes, hyperlipidemia, White blood cell count |
| Zhang et al. (b) | 2019 | China | NAFLD: n=4517, age, 42, 71.8%M  Control: n=19398, age, 38.8, 41.2%M | Validated FFQ | Abdominal ultrasound | Nut | Age, sex, BMI, moking status, alcohol drinking status, education level, occupation, household income, physical activity, family history of disease, history of hypertension, total energy intake, EPA + DHA intake, soft drinks intake and three main dietary pattern scores, triglycerides, low-density lipoprotein cholesterol, high-density lipoprotein cholesterol, fasting blood glucose and white blood cell count. |

(*Continued*)

**Supplementary Table 1** (*continued*)

| Author | Year | Location | Participant | Diagnosed method of NAFLD | Dietary measurement | Food groups | Confounder adjusted |
| --- | --- | --- | --- | --- | --- | --- | --- |
| Xia et al. | 2019 | China | NAFLD: n=7396, age 43.48, 74%M  Control: n=19818, age 38.47, 42%M | Validated FFQ | Abdominal ultrasound | fruits | Age, sex, BMI, smoking status, drinking status, education level, employment status, household income, physical activity, metabolic syndrome, total energy intake, intake of protein, carbohydrate, fat, EPA + DHA, fruit, vegetable, soft drink and juice, and family history of cardiovascular disease, hypertension, hyperlipidemia, and diabetes. |
| Chiu et al. | 2018 | Taiwan | Vegetarian group: n=1273, age 45±9, 78% F Non-vegetarians group: n=2127, age 54±10, 59% F | Validated FFQ | Abdominal ultrasound | Red meat,  fish,  dairy,  eggs,  legumes,  whole grains, refinedgrains, vegetables, fruits | Age, sex, education, smoking status, history of alcohol drinking, total energy intake, vegetarian diet, BMI |

(*Continued*)

**Supplementary Table 1** (*continued*)

| Author | Year | Location | Participant | Diagnosed method of NAFLD | Dietary measurement | Food groups | Confounder adjusted |
| --- | --- | --- | --- | --- | --- | --- | --- |
| Zelber-sagi et al. | 2018 | Israel | n=789, age: 58.83±6.58, 52.60% M | Validated FFQ | Abdominal ultrasound | Red meat | Age, gender, energy intake, BMI, physical activity, smoking status,weekly alcohol portions, saturated fat and cholesterol intake. |
| Baratta et al. | 2017 | Italy | n=584, age 56.2±12.4, 38.2% F | Validated Med-diet questionnaire | Abdominal ultrasound | Fruits, vegetavles, legumes, fish, whole grains | Age, gender, waist circumference, hypertriglyceridemia,arterial hypertension, statin use, ALT, Med-diet score |
| Meng et al. | 2018 | China | n=26790, age: 41.2±11.9, 50.5% M | Validated FFQ | Abdominal ultrasound | Soft drinks | Age, sex, BMI, smoking status, drinker status, educational level, employment status, household income, family history of diseases, total energy intake, protein intake, carbohydrate intake, fat intake, EPA and DHA intake, physical activity and metabolic syndrome, consumption of other beverages and Tianjin dietary pattern |
| Shim et al. | 2017 | South Korea | Case: n=58, age 49.32±13.84, 56.9% M Controls: n=81, age 49.40±16.11, 37.0% M | Validated FFQ | Abdominal ultrasound | Red meat, fish, vegetables | Gender, BMI |

(*Continued*)

**Supplementary Table 1** (*continued*)

| Author | Year | Location | Participant | Diagnosed method of NAFLD | Dietary measurement | Food groups | Confounder adjusted |
| --- | --- | --- | --- | --- | --- | --- | --- |
| Tajima et al. | 2016 | Japan | N=2444, age: 53.4±8.4, 40%M | BDHQ | Abdominal ultrasound | Refined grains, soft drinks | age, BMI, exercise, smoking status, total energy intake, alcohol intake, dietary fiber intake, EPA and DHA intake, coffee intake, and soft drink intake |
| Chan et al. | 2015 | HongKong | Case: n=220, age 51.0±9.3, 53.6% M Controls: n=677, age 47.0±10.8, 37.1% M | Validated FFQ | H-MRS | Soft drinks, eggs, fish, fruits, refined grains, nuts, red meat, legumes, vegetables, dairy | BMI, current smoker status, current drinker status, central obesity, triglyceride level, reduced HDL-cholesterol, hypertension, impaired fasting glucose or diabetes, and the PNPLA3 genotypes, energy intake |
| Ma et al. | 2015 | US | N=2634, age>35 | Validated FFQ | MDCT | Soft drinks | age, sex, BMI, energy intake, alcohol intake, dietary fiber, dietary fat, dietary protein, sugar-sweetened beverages or diet soda, smoking, and Framingham cohort. |

(*Continued*)

**Supplementary Table 1** (*continued*)

| Author | Year | Location | Participant | Diagnosed method of NAFLD | Dietary measurement | Food groups | Confounder adjusted |
| --- | --- | --- | --- | --- | --- | --- | --- |
| Jia et al. | 2015 | China | Case (M): n=1368, age 40.7±9.5; Case (F): n=1499, age 41.1±8.9  Controls (M): n=1037, age 44.0±7.3; Controls (F): n=302, age 51.1±7.1 | Validated FFQ | Abdominal ultrasound | Fruits, red meat, vegetables, soft drinks, refined grains | Age, body mass index, smoking status, drinking status, physical activity, metabolic syndrome, total energy intake, and family history of cardiovascular disease, hypertension, hyperlipidemia and diabetes |
| Zhou et al. | 2019 | China | M=1238 (Age 49.1±12.4), with NAFLD: 328 (age: 47.9±12.5) F=1928 (Age: 50.0±11.6), with NAFLD: 388 (Age: 51.8±11.4) | Validated FFQ | Abdominal CT | Red meat | Age, BMI, systolic blood pressure, HbA1c, postmenopausal status, annual house income, education level, intake, and alcohol consumption. |
| Tajima et al. | 2018 | Japan | M: n=997, age (fruits): 57.7±7.5, age (vegetables): 56.0±8.0 F: n=1467, age (fruits): 54.7±8.1, age (vegetables): 54.7±8.0 | BDHQ | Abdominal ultrasound | Fruits, vegetables | habitual exercise, current smoking, intakes of total energy, alcohol, EPA + DHA, coffee, soft drink, total vegetables, and fruit. |
| Zelber-sagi et al. | 2007 | Israel | n=349, age 50.7±10.4, 52.7% M | Validated FFQ | Abdominal ultrasound | Soft drinks, red meat, fish | Age, gender, BMI and total calories |

^1^ EPA, eicosapentaenoic acid; DHA, docosahexaenoid acid; FFQ, food frequency questionnaire; BDHQ, brief-type self-administrated diet questionnaire.

**Supplementary Table 2** Characteristics of case-control studies included in the meta-analysis^2^

| Author | Year | Location | Participant | Diagnosed method of NAFLD | Dietary measurement | Food groups | Confounder adjusted |
| --- | --- | --- | --- | --- | --- | --- | --- |
| Noureddin et al. | 2019 | US | NALFD: n=2974, age=57.7, 73.4%M  Control: n=29474, age=57.8, 37.7%M | Medicare claims | Validated QFFQ | Red meat, vegetales,  fruits | BMI, alcohol intake, coffee, intake, total  soda intake, vigorous physical activity, energy |
| Chen et al. | 2019 | China | NAFLD: n=534  Control: n=534  Age: 18-70 match with age | Abdominal ultrasound | Validated FFQ | Nut | Total energy intake, age, sex, income, smoking status, educational level, tea-drinking status, occupationanl status, marital status, BMI, physical activity, the history of diabetes, hypertension and hyperlipidaemia, MUFA and PUFA intake. |
| Bahrami et al. | 2019 | Iran | NAFLD: n=196, age 42.3±11.9, 51.5M  Control: n=803, age 43.5±14.5, 41%M | Abdominal ultrasound & fibroscan | Validated FFQ | legumes | Energy intake, BMI, physical activity, dyslipidemia, diabetes, smoking, dietary intake of fat, protein, carbohydrate, fruits, vegetables, and whole grain, dietary intake of red/processed meat and high-fat dairy |
| Kalafati et al. | 2018 | Greece | Case: n=134, age 50.36±10.51, 45.5% M Controls: n=217, age 43.75±11.23, 39.2% M | Abdominal ultrasound | Validated FFQ | Refined grains, dairy, fish, nuts | Age, gender, BMI or energy intake, pack-years and PAL |

(*Continued*)

**Supplementary Table 2** (*continued*)

| Author | Year | Location | Participant | Diagnosed method of NAFLD | Dietary measurement | Food groups | Confounder adjusted |
| --- | --- | --- | --- | --- | --- | --- | --- |
| Mokhtari et al. | 2017 | Iran | Case: n=169, age 42.65±12.21, 47.9% M; Controls: n=782, age 43.71±14.52, 40.2% M | Abdominal ultrasound & fibroscan | Validated FFQ | Eggs | Age, energy intake, BMI, history of diabetes, smoking, and physical activity |
| Katsagoni et al. | 2016 | Greece | Case: n=100, age 45.51±11.6, 72.0% M Controls: n=55, age: 44.82±12.06, 62.0% M | Abdominal ultrasound or liver biopsy | Validated FFQ | Nuts, vegetables | Age, sex, waist circumference, HOMA-IR, adiponectin, and TNF-a |
| Georgoulis et al. | 2014 | Greece | Case: n=58, age 44.5±11.6, 62.1% M Control: n=58, age 44.6±12.0, 62.1% M | Abdominal ultrasound and/or liver biopsy | Validated FFQ | Whole grains, refined grains | Sex, daily energy intake, abdominal fat level |
| Abid et al. | 2009 | Israel | NAFLD without MesS: n=29, age 41±11, 47% M  Control: n=30, age 40±10, 49% M | Abdominal ultrasound | Validated Block FFQ | Soft drinks | Age, sex, smoking habits, physical activity, dietary composition, body mass index, metabolic syndrome, triglyceride, HOMA, and metabolic biomarkers. |
| Miele et al. | 2014 | Italy | Case: n=234, age 62.06±13.98, 61% M Controls: n=349, age 44.94±14.11, 67% M | Abdominal ultrasound | Validated FFQ | Fruits, red meat, fish | Age, sex, drinking habits, fruit, and grilled meat and fish |

^2^ HOMA-IR, homeostasis model assessment for insulin resistance; FFQ, food frequency questionnaire; BDHQ, brief-type self-administrated diet questionnaire.

**Supplementary Table 3** Bias risk of each domain of included studies assessed by ROBINS-I

| Study | Bias due to confounding | Bias in selection of participants | Bias in measurement of interventions | Bias due to departures from intended interventions | Bias due to missing data | Bias in measurement of outcomes | Bias in selection of the reported result | Overall bias |
| --- | --- | --- | --- | --- | --- | --- | --- | --- |
| Georgoulis et al. 2014 | Low risk | Low risk | Low risk | Low risk | Low risk | Low risk | Low risk | Low risk |
| Chiu et al. 2018 | Moderate risk | Low risk | Low risk | Low risk | Low risk | Low risk | Low risk | Moderate risk |
| Zelber-Sagi et al. 2018 | Moderate risk | Low risk | Low risk | Low risk | Low risk | Low risk | Low risk | Moderate risk |
| Meng et al. 2018 | Moderate risk | Low risk | Low risk | Low risk | Low risk | Low risk | Low risk | Moderate risk |
| Shim et al. 2017 | Moderate risk | Low risk | Low risk | Low risk | Low risk | Low risk | Low risk | Moderate risk |
| Jia et al. 2015 | Moderate risk | Low risk | Low risk | Low risk | Low risk | Low risk | Low risk | Moderate risk |
| Zhang et al. 2019 (a) | Low risk | Low risk | Low risk | Low risk | Low risk | Low risk | Low risk | Low risk |
| Tajima et al. 2018 | Low risk | Low risk | Low risk | Low risk | Low risk | Low risk | Low risk | Low risk |

*(Continued)*

**Supplementary Table 3** *(Continued)*

| Study | Bias due to confounding | Bias in selection of participants | Bias in measurement of interventions | Bias due to departures from intended interventions | Bias due to missing data | Bias in measurement of outcomes | Bias in selection of the reported result | Overall bias |
| --- | --- | --- | --- | --- | --- | --- | --- | --- |
| Tajima et al 2016 | Low risk | Moderate risk | Low risk | Low risk | Low risk | Low risk | Low risk | Moderate risk |
| Baratta et al. 2017 | Low risk | Low risk | Low risk | Low risk | Low risk | Low risk | Low risk | Low risk |
| Chan et al. 2015 | Low risk | Low risk | Low risk | Low risk | Low risk | Low risk | Low risk | Low risk |
| Katsagoni et al. 2017 | Low risk | Low risk | Low risk | Low risk | Low risk | Low risk | Low risk | Low risk |
| Kalafati et al. 2018 | Low risk | Low risk | Low risk | Low risk | Low risk | Low risk | Low risk | Low risk |
| Ma et al. 2015 | Low risk | Low risk | Low risk | Low risk | Low risk | Low risk | Low risk | Low risk |
| Zhou et al. 2019 | Low risk | Low risk | Low risk | Low risk | Low risk | Low risk | Low risk | Low risk |
| Miele et al. 2014 | Low risk | Low risk | Low risk | Low risk | Low risk | Low risk | Low risk | Low risk |

*(Continued)*

**Supplementary Table 3** *(Continued)*

| Study | Bias due to confounding | Bias in selection of participants | Bias in measurement of interventions | Bias due to departures from intended interventions | Bias due to missing data | Bias in measurement of outcomes | Bias in selection of the reported result | Overall bias |
| --- | --- | --- | --- | --- | --- | --- | --- | --- |
| Zelber-Sagi et al.  2007 | Moderate risk | Low risk | Low risk | Low risk | Low risk | Low risk | Low risk | Moderate risk |
| Mokhtari et al. 2017 | Low risk | Low risk | Low risk | Low risk | Low risk | Low risk | Low risk | Low risk |
| Abid et al. 2009 | Moderate risk | Low risk | Low risk | Low risk | Low risk | Low risk | Low risk | Moderate risk |
| Bahrami et al. et al. 2019 | Low risk | Low risk | Low risk | Low risk | Low risk | Low risk | Low risk | Low risk |
| Xia et al. 2019 | Low risk | Low risk | Low risk | Low risk | Low risk | Low risk | Low risk | Low risk |
| Zhang et al. 2019 (b) | Low risk | Low risk | Low risk | Low risk | Low risk | Low risk | Low risk | Low risk |
| Chen et al. 2019 | Low risk | Low risk | Low risk | Low risk | Low risk | Low risk | Low risk | Low risk |
| Noureddin et al. 2019 | Low risk | Low risk | Moderate risk | Low risk | Low risk | Moderate risk | Low risk | Moderate risk |

**Supplementary Table 4** Subgroup analysis among cross-sectional studies of red meat and NAFLD, stratified by study type, geographic location, number of cases, and dietary assessment

| Dietary factor | No of studies | OR (95% CI) | P | Heterogeneity | |
| --- | --- | --- | --- | --- | --- |
|  |  |  |  | I^2^ | p |
| Red meat |  |  |  |  |  |
| Cross-sectional studies | 7 | 1.121 (1.042-1.207) | 0.002 | 48.7% | 0.069 |
| Low risk of bias | 2 | 1.218 (1.018-1.458) | 0.031 | 0.0% | 0.817 |
| Geographic location |  |  |  |  |  |
| Asia | 7 | 1.121 (1.042-1.207) | 0.002 | 48.7% | 0.069 |
| Number of cases |  |  |  |  |  |
| <1000 | 5 | 1.311 (1.142-1.504) | 0.000 | 0.0% | 0.415 |
| ≥1000 | 2 | 1.005 (0.967-1.150) | 0.227 | 0.0% | 0.340 |
| Dietary assessment |  |  |  |  |  |
| Validated | 6 | 1.244 (1.050-1.472) | 0.011 | 56.4% | 0.043 |
| Not validated | 1 | 1.196 (0.911-1.570) | 0.197 | NA | NA |

NA, not applicable; OR, Odd Risk

**Supplementary Table 5** Subgroup analysis among cross-sectional studies of soft drink and NAFLD, stratified by study type, geographic location, number of cases, and dietary assessment

| Dietary factor | No of studies | OR (95% CI) | P | Heterogeneity | |
| --- | --- | --- | --- | --- | --- |
|  |  |  |  | I^2^ | p |
| Soft drinks |  |  |  |  |  |
| Cross-sectional studies | 6 | 1.294 (1.191-1.406) | 0.000 | 25.3% | 0.245 |
| Low risk of bias | 2 | 1.575 (1.133-2.189) | 0.007 | 0.0% | 0.880 |
| Geographic location |  |  |  |  |  |
| Asia | 5 | 1.284 (1.180-1.397) | 0.000 | 29.9% | 0.222 |
| US | 1 | 1.610 (1.038-2.496) | 0.033 | NA | NA |
| Number of cases |  |  |  |  |  |
| <1000 | 4 | 1.321 (1.117-1.563) | 0.001 | 45.8% | 0.136 |
| ≥1000 | 2 | 1.286 (1.168-1.414) | 0.000 | 7.0% | 0.300 |
| Dietary assessment |  |  |  |  |  |
| Validated | 5 | 1.276 (1.169-1.393) | 0.000 | 30.6% | 0.218 |
| Not validated | 1 | 1.460 (1.127-1.891) | 0.004 | NA | NA |

NA, not applicable; OR, Odd Risk

**Supplementary Table 6** Subgroup analysis among cross-sectional studies of fruits and NAFLD, stratified by study type, geographic location, number of cases, and dietary assessment

| Dietary factor | No of studies | OR (95% CI) | P | Heterogeneity | |
| --- | --- | --- | --- | --- | --- |
|  |  |  |  | I^2^ | p |
| Fruits |  |  |  |  |  |
| Cross-sectional studies | 6 | 0.991 (0.844-1.163) | 0.907 | 68.0% | 0.008 |
| Low risk of bias | 3 | 0.823 (0.424-1.597) | 0.564 | 79.9% | 0.007 |
| Geographic location |  |  |  |  |  |
| Asia | 5 | 0.988 (0.836-1.168) | 0.877 | 74.4% | 0.004 |
| Europe | 1 | 0.919 (0.275-3.070) | 0.891 | NA | NA |
| Number of cases |  |  |  |  |  |
| <1000 | 3 | 0.651 (0.483-0.878) | 0.005 | 0.0% | 0.437 |
| ≥1000 | 3 | 1.090 (0.976-1.217) | 0.125 | 55.2% | 0.107 |
| Dietary assessment |  |  |  |  |  |
| Validated | 5 | 0.943 (0.781-1.139) | 0.544 | 72.2% | 0.006 |
| Not validated | 1 | 1.182 (0.908-1.538) | 0.213 | NA | NA |

NA, not applicable; OR, Odd Risk

**Supplementary Table 7** Subgroup analysis among cross-sectional studies of vegetables and NAFLD, stratified by study type, geographic location, number of cases, and dietary assessment

| Dietary factor | No of studies | OR (95% CI) | P | Heterogeneity | |
| --- | --- | --- | --- | --- | --- |
|  |  |  |  | I^2^ | p |
| Vegetables |  |  |  |  |  |
| Cross-sectional studies | 6 | 1.005 (0.976-1.035) | 0.725 | 50.0% | 0.075 |
| Low risk of bias | 2 | 0.574 (0.353-0.932) | 0.025 | 10.2% | 0.291 |
| Geographic location |  |  |  |  |  |
| Asia | 5 | 0.851 (0.683-1.060) | 0.150 | 60.0% | 0.040 |
| Europe | 1 | 1.023 (0.315-3.323) | 0.970 | NA | NA |
| Number of cases |  |  |  |  |  |
| <1000 | 4 | 0.696 (0.528-0.916) | 0.010 | 0.0% | 0.398 |
| ≥1000 | 2 | 1.101 (0.980-1.040) | 0.528 | 0.0% | 0.783 |
| Dietary assessment |  |  |  |  |  |
| Validated | 5 | 0.793 (0.587-1.072) | 0.132 | 59.8% | 0.041 |
| Not validated | 1 | 0.970 (0.728-1.292) | 0.835 | NA | NA |

NA, not applicable; OR, Odd Risk
